# A Biomarker Panel (Bioscore) Incorporating Monocytic Surface and Soluble TREM-1 Has High Discriminative Value for Ventilator-Associated Pneumonia: A Prospective Observational Study

**DOI:** 10.1371/journal.pone.0109686

**Published:** 2014-10-07

**Authors:** Vimal Grover, Panagiotis Pantelidis, Neil Soni, Masao Takata, Pallav L. Shah, Athol U. Wells, Don C. Henderson, Peter Kelleher, Suveer Singh

**Affiliations:** 1 Magill Department of Anaesthesia, Critical Care and Pain, Chelsea and Westminster Hospital National Health Service Foundation Trust, London, United Kingdom; 2 Immunology Section, Department of Medicine, Imperial College, London, United Kingdom; 3 Department of Surgery and Cancer, Imperial College, London, United Kingdom; 4 Department of Immunology, Imperial College Healthcare National Health Service Trust, London, United Kingdom; 5 Department of Respiratory Medicine, Chelsea and Westminster Hospital National Health Service Foundation Trust, London, United Kingdom; 6 Department of Respiratory Medicine, Royal Brompton & Harefield Hospitals National Health Service Foundation Trust, London, United Kingdom; Yale University, United States of America

## Abstract

**Introduction:**

Ventilator-associated pneumonia (VAP) increases mortality in critical illness. However, clinical diagnostic uncertainty persists. We hypothesised that measuring cell-surface and soluble inflammatory markers, incorporating Triggering Receptor Expressed by Myeloid cells (TREM)-1, would improve diagnostic accuracy.

**Methods:**

A single centre prospective observational study, set in a University Hospital medical-surgical intensive Care unit, recruited 91 patients into 3 groups: 27 patients with VAP, 33 ventilated controls without evidence of pulmonary sepsis (non-VAP), and 31 non-ventilated controls (NVC), without clinical infection, attending for bronchoscopy. Paired samples of Bronchiolo-alveolar lavage fluid (BALF) and blood from each subject were analysed for putative biomarkers of infection: Cellular (TREM-1, CD11b and CD62L) and soluble (IL-1β, IL-6, IL-8, sTREM-1, Procalcitonin). Expression of cellular markers on monocytes and neutrophils were measured by flow cytometry. Soluble inflammatory markers were determined by ELISA. A biomarker panel (‘Bioscore’), was constructed, tested and validated, using Fisher’s discriminant function analysis, to assess its value in distinguishing VAP from non VAP.

**Results:**

The expression of TREM-1 on monocytes (mTREM-1) and neutrophils (nTREM-1) and concentrations of IL-1β, IL-8, and sTREM-1 in BALF were significantly higher in VAP compared with non-VAP and NVC (p<0.001). The BALF/blood mTREM-1 was significantly higher in VAP patients compared to non-VAP and NVC (0.8 v 0.4 v 0.3 p<0.001). A seven marker Bioscore (BALF/blood ratio mTREM-1 and mCD11b, BALF sTREM-1, IL-8 and IL-1β, and serum CRP and IL-6) correctly identified 88.9% of VAP cases and 100% of non-VAP cases.

**Conclusion:**

A 7-marker bioscore, incorporating cellular and soluble TREM-1, accurately discriminates VAP from non-pulmonary infection.

## Introduction

Ventilator-associated pneumonia (VAP) remains a common complication of critical illness, affecting over 10% of intubated patients, prolonging ICU stay, with an estimated attributable mortality of 13% [1]–[3]. This is despite the introduction of health improvement strategies such as Ventilator care bundles, which have apparently reduced the incidence [4], [5], even though antibiotic prescriptions remain high for pulmonary sepsis in ICU [5]. Standardisation of diagnostic criteria for VAP is important for benchmarking, but no single best definition exists [6]. This in part has led to proposals for simplifying definitions into infective and non-infective ventilator associated complications [7].

Confirmatory diagnosis by microbiological culture is often too slow for clinical need, whilst even quantitative microbiological analysis is subject to the variations in the sampling site, or elusive despite other criteria being fulfilled [8]. Biomarkers may facilitate clinical confirmation and aid differentiation of pulmonary from non-pulmonary sepsis. This would allow earlier, targeted antibiotic intervention, direct clinicians’ decision-making for ‘antibiotic de-escalation’ regimens and potentially reduce selective pressure for multi-resistant bacteria [9], [10]. The role of inflammatory biomarkers including TREM-1 (Triggering Receptor Expressed on Myeloid Cells-1), IL-1, IL-6, IL-8, Procalcitonin (PCT) and more traditional indices, i.e. white cell count and CRP remains unclear. Only some show clinical diagnostic utility for VAP [11]–[14]. Differences in definitions of VAP patient populations, severity of disease, and assay techniques account for much of the conflicting data reported [15], [16]. Furthermore, failure of many studies to consider the dynamic relationships between soluble and cell surface inflammatory proteins (e.g.TREM-1), differential expression of inflammatory markers by neutrophils and monocytes, and compartmentalization of inflammatory immune responses at the site of tissue infection in reference to blood, are likely contributory factors.

The aim of this study was to determine if, and which combination of paired blood and bronchoalveolar lavage fluid (BALF) inflammatory biomarkers (soluble and cell surface based, including TREM-1), could correctly classify patients with VAP from ventilated patients without evidence of pulmonary sepsis.

## Materials and Methods

### Study participants

Informed, witnessed and written assent was obtained from a relative or designated carer for all ventilated patients. Written consent was obtained from all day case bronchoscopy patients. Ethical permission was obtained from the local institutional board (Barking and Havering Local Research Ethics Committee, Ilford, Essex, UK), through the National Research Ethics Service (NRES) of the United Kingdom 08/H0702/61.

The study sample was selected from patients hospitalized between Feb 2009 and Aug 2011 in the Intensive Care unit, and Lady Kilmarnock bronchoscopy suite of the Chelsea and Westminster Hospital NHS Foundation Trust, London, United Kingdom. Adult patients (>18 years) were recruited into the following 3 groups; ventilator associated pneumonia, ventilated controls without evidence of pulmonary sepsis or with non-pulmonary sepsis (non-VAP), and non-ventilated non-infected controls (NVC).

In accordance with the 2005 guidelines of the American Thoracic Society/Infectious Diseases Society of America, the criteria for diagnosis of VAP were evidence of new infiltrates on chest radiographs after 48 hours of endotracheal intubation and presence of at least 2 of the following: fever (temperature >38°C or higher than basal temperature), abnormal white cell count (≥10 000/µL or <4000/µL), and purulent respiratory tract secretions [2]. As per recommendations, BALF samples were collected via directed bronchoscopy, semi-quantitatively reported (SQ) and cultured for microorganisms [17].

The clinical pulmonary infection score (CPIS) defined VAP and non-VAP [17], [18]. Thus, VAP was predefined as CPIS >5 and positive BALF microbiology. Non-VAP was predefined as CPIS score <6 and negative microbiology. This was a modification of the original CPIS, by additionally incorporating SQ microbiological data. The patient cohorts comprised non-infected ventilated patients, or individuals with non-pulmonary infection (i.e. intra-abdominal, indwelling devices) confirmed on clinical, radiological and microbiological grounds. To control for the effects of mechanical ventilation on pulmonary inflammation, BALF and blood samples were obtained from a cohort of non-ventilated control patients (NVC) undergoing day case bronchoscopy for non-infective respiratory disorders (i.e., chronic obstructive pulmonary disease, COPD, interstitial lung disease, ILD, or solitary pulmonary nodules).

Comparison of CPIS with the European Hospitals in Europe Link for Infection Control through Surveillance programme (HELICS) criteria (PN4) revealed excellent concordance using the Cohen kappa statistic (0.95) [15], [19]. Two patients with VAP would have been classified as non-VAP using HELICS and one patient with non-VAP could possibly have been placed into the VAP cohort. Initial chest radiographic interpretation was that of the clinical investigators, with all radiographs being independently confirmed by a radiologist.

Data on exclusion criteria, and description of procedures for obtaining informed consent and for sampling, processing of BALF and blood and group classification are provided in an online supplement.

### Laboratory studies

Twenty two individual inflammatory markers were measured. In blood these consisted of six cell surface [3 monocytic and 3 neutrophilic (TREM-1, CD11b and CD62L)] and five soluble proteins sTREM-1, IL-6, PCT, CRP and the white cell count (WCC). In BALF, the same six cell surface markers were measured and five soluble proteins above the limit of detection for ELISA were sTREM-1, IL-1β, IL-6, IL-8 and PCT. Nine BALF/blood marker ratios were calculated.

Immunophenotypic analysis was performed on peripheral blood and BALF cell suspensions using 5-colour flow cytometry (Cytomics FC500 Beckman Coulter, Beckman-Coulter, Villepinte, France). Blood and BALF cells were isolated following standard centrifugation procedures, and washed in phosphate buffered saline/1% fetal calf serum (FCS). 100 µl aliquot cell suspensions were then stained with monoclonal antibodies for 30 minutes. Further details on monoclonal antibodies used, on instrumentation and software analysis are provided in an on line supplement. CD45 staining and side scatter properties were initially used to select CD14 and CD16 positive cells as markers of monocytes and neutrophils respectively. Isotype controls were used to delineate specific protein expression on the cell surface of inflammatory cells. Geometric mean fluorescent intensity (MFI) was used as an index of protein concentration expressed by a particular blood or BALF cell population. Details on measurement of cytokines and inflammatory mediators (sTREM-1, IL-1β, IL-6 and IL-8) and PCT are provided in an online supplement. Urea was determined by ELISA (Abcam, Cambridge, UK) and was used to correct for dilutional effects in BALF [20].

### Statistical Analysis

Anthropometric data was reported as medians and inter-quartile ranges. Differences between the groups for individual biomarkers were determined using the Kruskal-Wallis test followed by the Mann-Whitney U test with Dunn’s post-hoc correction for multiple analyses when there were any statistical differences between individual groups.

Fisher’s discriminant function analysis (FDA) was used to determine the optimal combination of biomarkers that could discriminate between VAP and non-VAP patient groups. A variable was entered into the “model” if the significance level of its F-value was <0.05 and was removed if the significance level was ≥0.05. The model was then used to classify each of the 91 cases into a diagnostic group. In order to check that the result of the biomarker model was not skewed by the presence of outlier data the model was internally validated by means of the leave-one-out method, which involves omitting a single observation from the original sample, and then using the remaining observations to assign the omitted case either to the VAP or non-VAP patient group.

The model was cross-validated by repeat random sub-sampling - by repeatedly (10 times) randomly assigning original cases into a training cohort (60% of original cases) to obtain new classification function coefficients for the analytes derived from the original model. The new function coefficients obtained were applied to a test cohort that consisted of the remaining cases (40%), to confirm the reliability of the model [21]–[24]. Further statistical information is available in an online supplement. All analysis was conducted using the SPSS v19 software package (SPSS, Chicago, IL, USA) and GraphPad Prism software (California, USA). Independent statistical analysis was performed.

## Results

### Study participants

Ninety one patients were recruited consecutively. There were 27 VAP, 33 non-VAP and 31 NVC patients (Table 1). There were no statistically significant differences between the groups with respect to age, sex, history of cigarette smoking, presence of chest x-ray infiltrates and APACHE II score. Twenty eight-day mortality was 3 deaths in the VAP group, 4 in the non-VAP group (none in the NVC). The majority of patients in the VAP and non-VAP groups were receiving antibiotics at the time of sampling. Thirty percent of ventilated patients received steroids for sepsis. The distribution of steroids between VAP and non-VAP groups were not statistically significant. Nine VAP and 13 non-VAP patients were post-operative cases. Within the NVC group, 7 patients had lung cancer, 9 COPD, 2 pulmonary sarcoidosis, 1 lung fibrosis, 6 with benign lung nodules and 7 with normal findings.

**Table 1:** Characteristics of patients recruited to study.

	VAP	Non-VAP	NVC
Number of patients	27	33	31
Age	68 (23–84)	62 (18–89)	59 (18–84)
Sex (% male/% female)	70/30	52/48	61/39
CPIS	7 (6–9)	3 (0–5)	N/A
Microbiology (% +ve)	100	12	0
APACHE II score	18 (5–45)	15 (2–24)	N/A
Smoking (% current/ex/none)	44/15/40	30/21/49	35/13/52
Antibiotics (% pre-BALF)	89	70	32
CXR (% with shadowing)	96	55	81
Steroids (%)	30	30	6
28-day mortality (%)	11	12	0
Post-surgical (%)	37	39	0
Burns injury (% of cases)	15	15	0
WCC (x10^9^/l)	15 (4–24)	9 (3–27)	7 (3–18)*
CRP (mg/L)	84 (7–320)	102(2–341)^†^	6 (1–296)*

The median and range (lowest-highest) is shown for each group. APACHE II and CPIS are only applicable to the ventilated patients. Some variables are presented as percentages. Statistically significant differences between the groups were determined using the Mann-Whitney U test with post-hoc Dunn correction and are indicated as follows: VAP versus NVC (p<0.001)* and non-VAP versus NVC (p<0.001)^†^. CPIS = Clinical Pulmonary Infection Score. APACHE II = Acute Physiology and Chronic Health Evaluation II score. VAP = ventilator-associated pneumonia. NVC = non-ventilated control. Non-VAP = ventilated non-pulmonary infected control. CXR = Chest X-ray. WCC = White cell count. CRP = C-reactive protein.

The following organisms were isolated (patients): Serratia marcescens (2), Klebsiella spp (4), Pseudomonas spp (9), methicillin sensitive staphylococcal aureus, MSSA (4), methicillin resistant staphylococcal aureus, MRSA (3), Escherischia coli, (5), Acinetobacter baumanii (5), Stenotrophomonas (2) and Proteus mirabilis (2). Twenty eight organisms were isolated from VAP patients and the remaining eight bacteria were found in non-VAP patients (non-pulmonary infection).

The CRP was significantly elevated in VAP and non-VAP compared to NVC group (p<0.001). White cell count was significantly higher in VAP than NVC (p<0.001). Neither CRP nor WCC distinguished VAP from non-VAP.

### Cellular and soluble inflammatory mediators in blood

In blood, there was no significant difference in the expression of cellular and soluble biomarkers between VAP and non-VAP (Table 2). However, the concentration of sTREM-1, IL-6, PCT and expression of CD62L on CD14 gated monocytes were significantly higher in VAP and non-VAP groups compared with NVC (Table 2). This suggests blood based biomarker activation resulting from ventilation, but that it is not discriminatory between VAP and non-VAP patients.

**Table 2:** Expression of cell-surface and soluble proteins in study participants with VAP, non-VAP and NVC.

	VAP	Non-VAP	NVC
**Blood**			
mTREM-1	5.1 (3.2–8.6)	4.6 (3.1–6.1)	6.5 (4.3–10.9)
nTREM-1	4.7 (2.6–7.3)	3.8 (2.3–6.1)	4.5 (3.1–7.4)
mCD11b	47.2 (30.0–70.0)	43.3 (27.6–52.3)	39.2(21.7–51.8)
nCD11b	44.0 (33.4–91.9)	59.8 (43.4–82.9)	49.0 (38.0–81.0)
mCD62L	9.4 (7.3–15.1)	9.5 (7.4–13.2)	5.4 (3.9–9.4)*
nCD62L	9.6 (6.0–17.0)	8.3 (6.0–10.5)	8.6 (6.8–10.5)
sTREM-1 (µg/ml)	0.18 (0.01–0.03)	0.15 (0.08–0.30)	0.09 (0.06–0.15)^†,‡^
IL-1β (µg/ml)	N/A	N/A	N/A
IL-6 (µg/ml)	0.09 (0.03–0.21)	0.08 (0.03–0.17)	0.008 (0.005–0.02)^*^
IL-8 (µg/ml)	N/A	N/A	N/A
PCT (ng/ml)	1.3 (0.3–5.3)	2.9 (0.6–8.3)	N/A^*^
**Corrected BALF**			
mTREM-1	3.9 (2.5–5.4)	1.6 (1.1–2.3)	1.8 (1.2–2.9)^§^
nTREM-1	2.0 (1.7–3.3)	1.5 (1.2–2.2)**	1.7 (1.3–3.0)
mCD11b	25.2 (9.0–81.2)	18.6 (13.7–31.2)	21.0 (6.9–47.3)
nCD11b	47.0 (15.1–86.0)	32.9 (20.3–62.5)	24.0 (6.0–73.5)
mCD62L	1.2 (1.0–1.5)	1.1 (1.0–1.3)	1.2 (1.0–1.4)
nCD62L	1.4 (1.0–2.1)	1.1 (1.0–1.4)	1.2 (1.0–1.7)
sTREM-1 (µg/ml)	20.14 (9.45–43.94)	5.19 (2.83–10.96)^††^	7.61 (3.05–18.32)
IL-1β (µg/ml)	3.02 (1.47–8.59)	0.79 (0.36–1.51)	0.53 (0.19–2.79)^§^
IL-6 (µg/ml)	3.80 (1.32–17.71)	2.08 (1.23–5.75)	1.45 (0.52–2.52)^‡‡^
IL-8 (µg/ml)	48.60 (20.78–101.10)	12.16(5.71–17.3)^§§^	16.33 (3.12–67.15)
PCT (ng/ml)	16.8 (9.7–51.7)	12.5(6.8–27.4)	9.6(4.1–18.2)
**Corrected BALF/blood ratio**			
mTREM-1	0.8(0.5–1.0)	0.4(0.2–0.5)	0.3(0.2–0.4)^§^
nTREM-1	0.6(0.2–0.8)	0.4(0.3–0.8)	0.4(0.2–1.1)
mCD11b	0.53(0.4–2.3)	0.4(0.2–0.7)^§§^	0.5(0.2–1.3)
nCD11b	0.7(0.5–2.0)	0.5(0.2–0.9)	0.5(0.1–1.4)
mCD62L	0.2(0.1–0.5)	0.1(0.1–0.2)	0.2(0.1–0.3)
nCD62L	0.2(0.1–0.3)	0.2(0.1–0.2)	0.2(0.1–0.2)
sTREM-1	190(70–337)	30(11–85)^§§^	84(26–228)^ll^
IL-1β	N/A	N/A	N/A
IL-6	77(20–145)	43(41–230)	134(230–355)
IL-8	N/A	N/A	N/A
PCT	29(3–55)	4(2–23)	N/A

The median and interquartile range for each patient group is reported. Statistically significant differences between groups were determined using the Mann-Whitney U and post hoc Dunn correction as follows: VAP and non-VAP versus NVC (p<0.001)^*^, VAP versus NVC (p<0.001)^†^ and non-VAP versus NVC (p<0.05)^‡^, VAP versus non-VAP and NVC (p<0.001)^§^, VAP versus non-VAP (p<0.01)^**^, VAP versus non-VAP (p<0.001)^††^, VAP versus NVC (p<0.01)^‡‡^, VAP versus non-VAP (p<0.001)^§§^ and NVC>non-VAP (p<0.01)^ll^. The lower limits of detection for the sTREM-1, IL-1β, IL-6, IL-8 and PCT assays were 0.01 µg/ml, 0.001 µg/ml, 0.0007 µg/ml, 0.004 µg/ml and 0.05 ng/ml respectively. N/A indicates below assay detection limit. BALF levels were corrected for dilution occurring with bronchoscopy using urea analysis. BALF/blood ratios were only calculable if BALF and blood measurements were obtained. VAP = ventilator-associated pneumonia. Non-VAP = ventilated patients with no evidence of pulmonary infection. NVC = non-ventilated non-infected patients.

### Cellular and soluble inflammatory mediators in BALF

**Figure 1:**
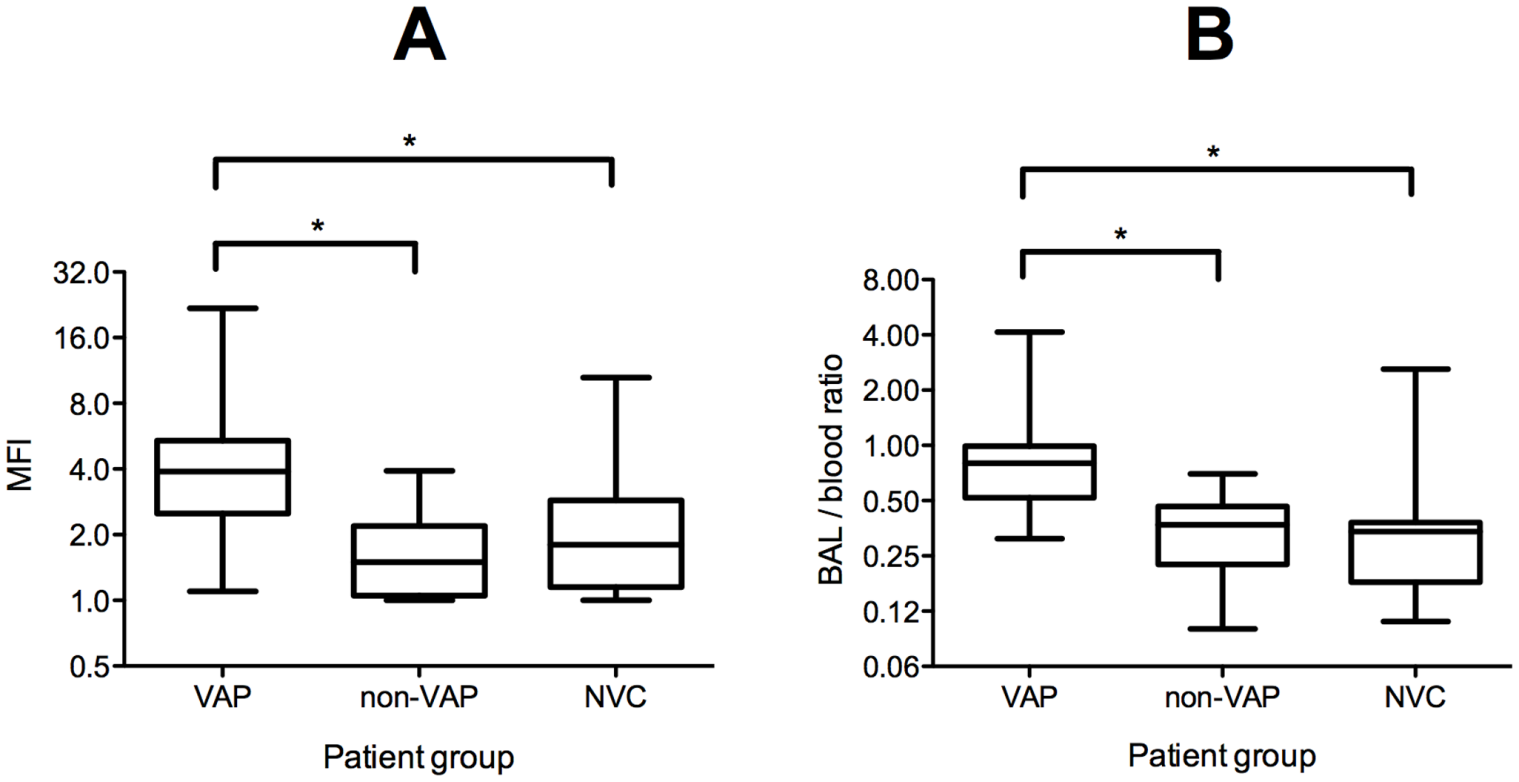
BALF levels and BALF/blood ratios of monocytic TREM-1. Box (interquartile) and whisker (range) plots showing expression of TREM-1 by CD14+ monocytes in BALF (Figure 1a) and the BALF/blood ratio of TREM-1 expression by monocytes in blood and BALF (Figure 1b) from patients with VAP, non-VAP (ventilated non-pulmonary infected control) and NVC (non-ventilated control). BALF levels were corrected for dilution occurring with bronchoscopy using urea measurement. Statistically significant differences between groups were determined using the Mann-Whitney U and post hoc Dunn correction as follows: monocyte TREM-1 levels for VAP versus non-VAP and NVC (p<0.001)* and BALF/blood monocytic TREM-1 ratio VAP versus non-VAP and NVC (p<0.001)*. MFI = mean fluorescence intensity.

**Figure 2:**
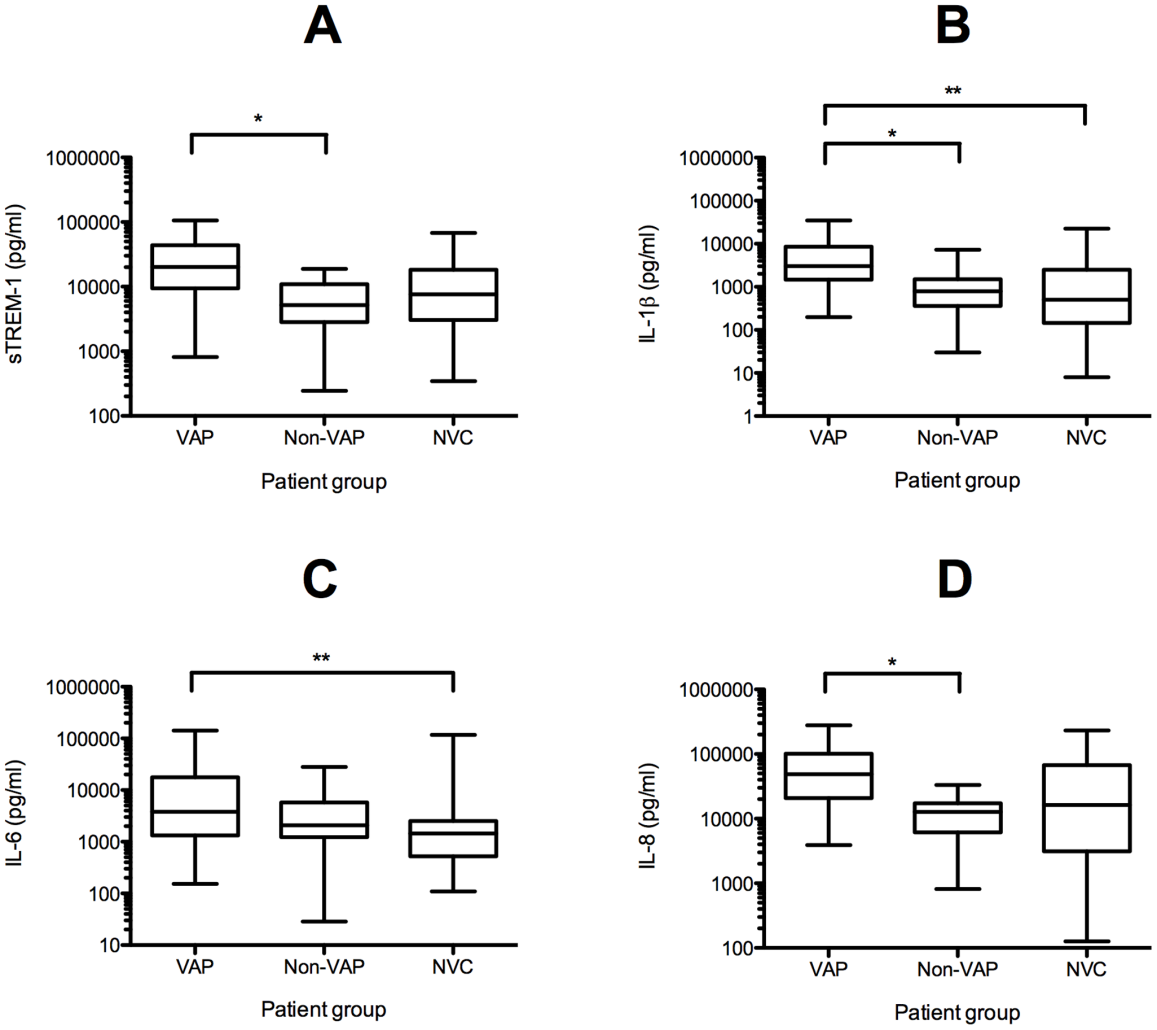
Soluble cytokine levels in BALF. Box (interquartile) and whisker (range) plots showing (a) sTREM-1, (b) IL-1β, (c) IL-6 and (d) IL-8 levels in BALF of patients with VAP, non-VAP (ventilated non-pulmonary infected control) and NVC (non-ventilated control). The BALF levels were corrected for dilution occurring with bronchoscopy using urea measurement. The concentration of BALF sTREM-1, IL-1β and IL-8 were significantly higher in VAP than non-VAP (p<0.001)*. BALF IL-1β and IL-6 were higher in the VAP compared with the NVC patient group (p<0.001)**.

By contrast, analysis of BALF showed significantly increased expression of cellular mTREM-1 and nTREM-1, and increased concentration of soluble IL-1β in VAP compared with non-VAP and NVC groups (p<0.001) (Table 2). Furthermore, whilst the increased expression of mTREM-1 from BALF in VAP was significant (p<0.001) (Figure 1a), this difference between VAP and the other two groups was greater when the compartmentalization ratio BALF/blood mTREM-1 was used (Figure 1b). This was not the case for BALF/Blood nTREM-1. The BALF/Blood ratios of CD11b on monocytes and sTREM-1 were also significantly higher in VAP group compared to non-VAP, but not when compared to NVC (Table 2). The expression of cellular mTREM-1, nTREM-1, and CD11b was lower in BALF than blood (Table 2), although the reductions seen were notably less in patients with VAP, as compared with non-VAP and NVC groups, hence the higher BALF/blood ratio (Table 2). Other soluble markers IL-1β, IL**-**8 and sTREM-1 were significantly raised in the VAP compared with non-VAP groups. IL-6 was similar in VAP and non-VAP groups but higher than NVC (Figure 2). None of the individual markers in blood, BALF or BALF/blood ratios had sufficient accuracy in distinguishing VAP from non-VAP (data not shown).

### Classification of individual cases within each study group using a biomarker panel

To determine whether a biomarker panel might have better discriminating ability than individual markers, and to separate the effects of ventilation from infection, Fisher Discriminant Analysis (FDA) was performed to build a ‘model’ that could best predict to which group (VAP and non-VAP) a study participant belonged on the basis of the biological measurements alone. To build the model we used all the VAP and non-VAP cases in the study and the 22 different markers and their compartmentalized ratios. A seven marker Bioscore consisting of BALF/blood cell expression ratio for monocyte mTREM-1 and mCD11b, BALF levels of sTREM-1, IL-8 and IL-1β, blood levels of CRP and IL-6 was shown to discriminate between VAP and non-VAP patients.

The 7 marker-bioscore produced 100% correct classification of the non-VAP patients and 88.9% correct classification of VAP patients. The NVC group which was treated as an unknown was defined as non-VAP in 90.7% of the cases using this model. In order to control for the possibility that the findings of the biomarker panel might be skewed by results obtained from any particular patient, we performed a leave one out cross validation analysis which produced the same level of accuracy with the original model (100% for non-VAP, 88.9% for VAP).

In order to assess the robustness of the model further, individual cases were then randomly assigned into a training cohort (60% of original cases) to obtain new classification function coefficients for the 7 analytes and the remaining 40% were used as unknowns for classification. In this cross-validation model the average predictive accuracies for the patients in the testing cohort were 71.0% for VAP and 98.5% for non-VAP. The reduction in classification for VAP was largely driven by the model attributing a number of NVC as VAP and to the limited power of the testing cohort analysed.

## Discussion

This study demonstrates that a combination of cell surface and soluble markers of inflammation, in particular TREM-1, sampled in blood and BALF simultaneously, can accurately discriminate VAP from ventilated patients without pulmonary sepsis. The use of a compartmentalization ratio, as a measure of site-specific immune response, results in a further improvement in diagnostic classification. These data have implications for the accurate diagnosis [25], antibiotic usage and management of VAP [26]. The results also address a potential weakness of previous studies which have measured only soluble mediators, often in one compartment. These may not fully account for the dynamic interaction between cell surface receptors and their soluble counterparts (e.g. mTREM-1 and sTREM-1 respectively), and site specific flux between the alveolar lung space and blood [27].

The findings suggest that monocytic surface receptor mTREM-1 and its neutrophilic counterpart nTREM-1 are compartmentalized within the lung, with increased expression in VAP. Although the expression of TREM-1 on pulmonary inflammatory cells has not to our knowledge previously been assessed in patients with VAP, the results are consistent with increased mTREM-1 reported in patients with community acquired pneumonia [28]. Soluble TREM-1 levels in BALF were significantly elevated and discriminatory in patients with VAP compared to non-VAP, in keeping with some, [12], [14], [29], [30] but not all studies, [13], [31], [32]. The BALF/blood ratio of mTREM-1, mCD11b and sTREM-1 were significantly higher in patients with VAP compared to those without VAP suggesting site-specific utility. Pulmonary infection may be distinguished from abdominal infection by combining BALF sTREM-1 and blood Procalcitonin measurement, although with lesser discrimination than our use of combined cell surface/soluble markers [12]. Ramirez et al reported the discriminative ability of site-sampled sTREM-1 for identifying pulmonary from abdominal infection in a critically ill cohort [33]. Thus, analysis of site-specific inflammatory markers may be useful in distinguishing infective sites, although measurement of cell surface markers over soluble proteins will not be influenced by dilutional variance from BALF.

The BALF/blood ratios of neutrophil-based nTREM-1 and nCD11b were not raised in VAP, unlike their monocytic counterparts. This difference is consistent with data from patients in septic shock, in whom blood mTREM-1 but not nTREM-1 levels increased compared with controls [34]. Expression of TREM-1 on neutrophils initially falls over minutes and then increases following in vitro LPS stimulation. In contrast TREM-1 levels on monocytes steadily increase over hours. It is possible that the recruitment timescale for VAP misses early neutrophilic changes [35]. Indeed, expression of TREM-1 by neutrophils may have passed its peak before time definitions of VAP allow measurement. Sampling individuals with suspected VAP at earlier time points may clarify differential kinetics of TREM-1 expression.

Surface TREM-1 may act as a link in the pathway from infective organism, to upregulation of the inflammatory cytokines. That said, it is unlikely to be specific for infection as opposed to inflammation. Experimentally, mTREM-1 activation in conjunction with lipopolysaccharride (LPS) increases IL-8 and IL-1β release, with amplification seen in septic shock, as in our study, [36], [37]. Such changes in IL-8 and IL-1β are in accordance with Conway-Morris et al, who have demonstrated high area under the curve (AUC) for them in suspected VAP [13]. Others have found elevated BALF IL-1β and IL-6 levels in VAP, when using a lung to blood ratio like in this study [38]. The BALF/blood ratios of soluble cytokines were non-discriminatory in this study, perhaps due to significant compartmentalization by the time of sampling, producing very low blood levels.

The difference in expression of mTREM-1 in VAP from non-VAP was more notable in BALF than blood, although the MFI were lower in BALF (Table 2). This may be due to increased shedding of up-regulated BALF mTREM-1 within the lung, as evidenced by the significantly greater increase in soluble sTREM-1 compared with blood. A potential mechanism is suggested to involve the balance between bacterial induced metalloproteinase (MMP) mediated cleavage of TREM-1 from surface of monocytes and action of specific MMP inhibitors [39]. Moreover, neutrophil derived MMP production is seen to increase markedly in BALF as compared to plasma from patients with hospital acquired pneumonia, whereas the specific tissue inhibitors of MMP (TIMP) were increased in plasma compared to BALF [40].

The limitations of this study are addressed here. First, no gold standard for VAP diagnosis exists. We included patients, who based on the CPIS scoring system, plus semi-quantitative microbiological testing were highly likely to have the presence or absence of VAP in order to test putative biomarkers. CPIS has been criticized by some for its potentially low diagnostic accuracy based upon clinico-radiological criteria. However, all current definitions of VAP remain subject to limitations. We acknowledge that semi-quantitative microbiology is less specific than Quantitative, but as sensitive for identifying pulmonary infection [41]. However, even the use of quantitative microbiology from directed BALF will potentially miss an important group of VAP patients (defined by standard clinic-patho-radiological criteria), if not meeting the predefined cutoff values of colony forming units/ml (cfu/ml). The arbitrary 48 h requirement for mechanical ventilation in most definitions of VAP provides important standardization but will also miss some pulmonary sepsis in ventilated patients developing prior to that timepoint. In order to mitigate against these concerns, we redefined the VAP and non VAP patients by other well established validated international criteria. Thus, the diagnostic definitions used were highly concordant when the HELICS criteria for pneumonia were used [15]. Reassuringly, from a biological perspective, the raised BALF IL-1β and IL-8 levels in VAP from our study concur with a group utilizing different diagnostic methodology, implying the validity to such approaches [13]. Therefore, the type of diagnostic criteria, particularly quantitative microbiology, used were not a major influence on the bioscore’s discriminability.

Second, this study did not encompass the whole range of infective aetiologies. For instance, no patients had mycoplasma, Legionella or proven respiratory viral pneumonias, and few had bilateral lung infiltrates. The management of viral pneumonias in particular, would benefit from early diagnostic biomarkers. Third, the absence of patients with ARDS does not allow us to comment on how the bioscore might perform in discriminating severe pulmonary inflammation from pulmonary infection. Fourth, and from a practical consideration, flow cytometry is a specialized technique. It requires samples with sufficient numbers of cells, which mandates adequate directed BALF samples, likely targeted bronchoscopy and makes serial biomarker analysis challenging. However, BALF samples are the current standard of care a microbiological diagnosis of VAP [16]. Fifth, a number of patients were receiving antibiotics and steroids at the time of sampling, with potential immunomodulatory activity. Given the prevalence of these key standard interventions in critically ill patients, we believe this pragmatic approach enhances the applicability of the findings. Sixth, the immune response to infecting pathogens in VAP, as in sepsis, is likely to involve neutrophils, monocytes and lymphocytes, [42]. As such we have not necessarily looked at all potentially relevant phagocytic or T cell markers. That said, the value of biomarker panels that include sTREM-1, PCT and CD64 on neutrophils has recently demonstrated the ability to predict sepsis in the setting of unselected critical illness, confirming the need to pursue such discriminatory panels in VAP, as in other disease states [42], [43]. Finally, we acknowledge that these results require validation in an external cohort of patients with suspected VAP, to see whether the bioscore 7 panel retains its discriminatory accuracy. Moreover, such a study is needed to see how the biomarker components, particularly mTREM-1, sTREM-1, IL1 and IL8, will perform and whether the BALF/blood ratio offers improved accuracy.

In conclusion, a 7 biomarker panel comprising soluble and cell-surface inflammatory markers including TREM-1 in combination with BALF/blood ratio differentiates VAP from non-pulmonary infection with good diagnostic accuracy. Such an approach, that incorporates these practically relevant and easily measurable biomarkers, to confirm or refute suspected VAP, requires confirmation.

## Supporting Information

Table S1 **BALF bacterial growth in patient groups.** The table shows the BALF microbiological results with patients of CPIS >6, CPIS <6 and CPIS = 6. CPIS = Clinical pulmonary infection score. VAP = Ventilator-associated pneumonia
(DOC)

Materials and Methods S1 **Supporting information on study participants, biomarker measurement, biomarker panel construction and validation.**
(DOC)

## References

[1] Alberti C, Brun-Buisson C, Burchardi H, Martin C, Goodman S, et al (2002) Epidemiology of sepsis and infection in ICU patients from an international multicentre cohort study. Int Care Med 28: 108–121. 10.1007/s00134-001-1143-z 11907653

[2] American Thoracic Society and Infectious Diseases Society of America (2005) Guidelines for the management of adults with hospital-acquired, ventilator-associated, and healthcare-associated pneumonia. Am J Respir Crit Care Med 171: 388–416. 15699079 10.1164/rccm.200405-644ST

[3] Melsen WG, Rovers MM, Groenwold RHH, Bergmans DCJJ, Camus C, et al (2013) Attributable mortality of ventilator-associated pneumonia: a meta-analysis of individual patient data from randomised prevention studies. Lancet Infect Dis 13: 665–71. 23622939 10.1016/S1473-3099(13)70081-1

[4] Bekaert M, Timsit JF, Vansteelandt S, Depuydt P, Vesin A, et al (2011) Attributable mortality of ventilator-associated pneumonia: a reappraisal using causal analysis. Am J Respir Crit Care Med 184: 1133–9. 21852541 10.1164/rccm.201105-0867OC

[5] Kollef MH, Hamilton CW, Ernst FR (2012) Economic impact of ventilator-associated pneumonia in a large matched cohort. Infect Control Hosp Epidemiol 33: 250–6. 22314062 10.1086/664049

[6] Klompas M (2007) Does this patient have ventilator-associated pneumonia? JAMA 297: 1583–93. 17426278 10.1001/jama.297.14.1583

[7] Kollef MH (2012) Prevention of ventilator-associated pneumonia or ventilator-associated complications: a worthy, yet challenging, goal. Crit Care Med 40: 271–7. 21946659 10.1097/CCM.0b013e318232e41d

[8] Chastre J, Trouillet JL, Combes A, Luyt CE (2010) Diagnostic techniques and procedures for establishing the microbial etiology of ventilator-associated pneumonia for clinical trials: the pros for quantitative cultures. Clin Infect Dis 51 Suppl 1 S88–92. 20597677 10.1086/653054

[9] Rea-Neto A, Youssef NC, Tuche F, Brunkhorst F, Ranieri VM, et al (2008) Diagnosis of ventilator-associated pneumonia: a systematic review of the literature. Crit Care 12: R56. 18426596 10.1186/cc6877 PMC2447611

[10] Lisboa T, Rello J (2008) Diagnosis of ventilator-associated pneumonia: is there a gold standard and a simple approach? Curr Opin Infect Dis 21: 174–8. 18317042 10.1097/QCO.0b013e3282f55dd1

[11] Gibot S, Cravoisy A, Levy B, Bene MC, Faure G, et al (2004) Soluble triggering receptor expressed on myeloid cells and the diagnosis of pneumonia. N Engl J Med 350: 451–8. 14749453 10.1056/NEJMoa031544

[12] Gibot S, Cravoisy A, Dupays R, Barraud D, Nace L, et al (2007) Combined measurement of procalcitonin and soluble TREM-1 in the diagnosis of nosocomial sepsis. Scand J Infect Dis 39: 604–8. 17577825 10.1080/00365540701199832

[13] Conway Morris A, Kefala K, Wilkinson TS, Moncayo-Nieto OL, Dhaliwal K, et al (2010) Diagnostic importance of pulmonary interleukin-1 beta and interleukin-8 in ventilator-associated pneumonia. Thorax 65: 201–7. 19825784 10.1136/thx.2009.122291 PMC2866736

[14] Anand NJ, Zuick S, Klesney-Tait J, Kollef MH (2009) Diagnostic implications of soluble triggering receptor expressed on myeloid cells-1 in BALF fluid of patients with pulmonary infiltrates in the ICU. Chest 135: 641–7. 18849395 10.1378/chest.08-1829

[15] Suetens C, Savey A, Labeeuw J, Morales I (2002) The ICU-HELICS programme: towards European surveillance of hospital-acquired infections in intensive care units. Euro Surveill 7: 127–8. 12631929 10.2807/esm.07.09.00359-en

[16] Conway Morris A, Kefala K, Simpson AJ, Wilkinson TS, Everingham K, et al (2009) Evaluation of the effect of diagnostic methodology on the reported incidence of ventilator-associated pneumonia. Thorax 64: 516–22. 19213771 10.1136/thx.2008.110239

[17] Pugin J, Auckenthaler R, Mili N, Janssens JP, Lew PD, et al (1991) Diagnosis of ventilator-associated pneumonia by bacteriologic analysis of bronchoscopic and nonbronchoscopic “blind” bronchoalveolar lavage fluid. Am Rev Respir Dis 143: 1121–1129. 2024824 10.1164/ajrccm/143.5_Pt_1.1121

[18] Luna CM, Blanzaco D, Niederman MS, Matarucco W, Baredes NC, et al (2003) Resolution of ventilator-associated pneumonia: prospective evaluation of the Clinical Pulmonary Infection Score as an early clinical predictor of outcome. Crit Care Med 31: 676–682. 12626968 10.1097/01.CCM.0000055380.86458.1E

[19] Cohen J (1960) A coefficient of agreement for nominal scales. Educ Psychol Meas 20: 37–46.

[20] Rennard SI, Basset G, Lecossier D, O’Donnell KM, Pinkston P, et al (1986) Estimation of volume of epithelial lining fluid recovered by lavage using urea as marker of dilution. J Appl Physiol 60: 532–8. 3512509 10.1152/jappl.1986.60.2.532

[21] Beirne P, Pantelidis P, Charles P, Wells AU, Abraham DJ, et al (2009) Multiplex immune serum biomarker profiling in sarcoidosis and systemic sclerosis. Eur Resp J 34: 1376–82. 10.1183/09031936.00028209 19541722

[22] Picard R, Cook D (1984) Cross-Validation of Regression Models. J Am Stat Assoc 79 575–583.

[23] Kohavi R (1995) A study of cross-validation and bootstrap for accuracy estimation and model selection. Proc of the Fourteenth Int Joint Conf on Art Intel 2: 1137–1143.

[24] Efron B, Tibshirani R (1997) Improvements on cross-validation: The.632+ Bootstrap Method. J Am Stat Assoc 92: 548–560.

[25] Chastre J, Fagon JY (2002) Ventilator-associated pneumonia. Am J Respir Crit Care Med 165: 867–903. 11934711 10.1164/ajrccm.165.7.2105078

[26] Leone M, Garcin F, Bouvenot J, Boyadjec I, Visintini P, et al (2007) Ventilator-associated pneumonia: breaking the vicious circle of antibiotic overuse. Crit Care Med 35: 379–385. 17205011 10.1097/01.CCM.0000253404.69418.AA

[27] Gibot S, Massin F, Le Renard P, Bene MC, Faure GC, et al (2005) Surface and soluble triggering receptor expressed on myeloid cells-1: expression patterns in murine sepsis. Crit Care Med 33: 1787–93. 16096457 10.1097/01.ccm.0000172614.36571.75

[28] Richeldi L, Mariani M, Losi M, Maselli F, Corbetta L, et al (2004) Triggering receptor expressed on myeloid cells: role in the diagnosis of lung infections. Eur Respir J 24: 247–50. 15332392 10.1183/09031936.04.00014204

[29] Huh JW, Lim CM, Koh Y, Oh YM, Shim TS, et al (2008) Diagnostic utility of the soluble triggering receptor expressed on myeloid cells-1 in bronchoalveolar lavage fluid from patients with bilateral lung infiltrates. Crit Care 12: R6. 18205941 10.1186/cc6770 PMC2374623

[30] Determann RM, Millo JL, Gibot S, Korevaar JC, Vroom MB, et al (2005) Serial changes in soluble triggering receptor expressed on myeloid cells in the lung during development of ventilator-associated pneumonia. Int Care Med 31: 1495–500. 10.1007/s00134-005-2818-7 16195904

[31] Oudhuis GJ, Beuving J, Bergmans D, Stobberingh EE, ten Velde G, et al (2009) Soluble Triggering Receptor Expressed on Myeloid cells-1 in bronchoalveolar lavage fluid is not predictive for ventilator-associated pneumonia. Int Care Med 35: 1265–70. 10.1007/s00134-009-1463-y PMC2698974 19343323

[32] Song Y, Lynch SV, Flanagan J, Zhuo H, Tom W, et al (2007) Increased plasminogen activator inhibitor-1 concentrations in bronchoalveolar lavage fluids are associated with increased mortality in a cohort of patients with Pseudomonas aeruginosa. Anesthesiology 106: 252–61. 17264718 10.1097/00000542-200702000-00012

[33] Ramirez P, Kot P, Marti V, Gomez MD, Martinez R, et al (2011) Diagnostic implications of soluble triggering receptor expressed on myeloid cells-1 in patients with acute respiratory distress syndrome and abdominal diseases: a preliminary observational study. Crit Care 2011: R50. 10.1186/cc10015 PMC3221980 21294874

[34] Gibot S, Le Renard PE, Bollaert PE, Kolopp-Sarda MN, Bene MC, et al (2005) Surface triggering receptor expressed on myeloid cells 1 expression patterns in septic shock. Int Care Med 31: 594–7. 10.1007/s00134-005-2572-x 15754199

[35] Knapp S, Gibot S, de Vos A, Versteeg HH, Colonna M, et al (2004) Cutting edge: expression patterns of surface and soluble triggering receptor expressed on myeloid cells-1 in human endotoxemia. J Immunol 173: 7131–4. 15585833 10.4049/jimmunol.173.12.7131

[36] Bleharski JR, Kiessler V, Buonsanti C, Sieling PA, Stenger S, et al (2003) A role for triggering receptor expressed on myeloid cells-1 in host defense during the early-induced and adaptive phases of the immune response. J Immunol 170: 3812–8. 12646648 10.4049/jimmunol.170.7.3812

[37] Bouchon A, Facchetti F, Weigand MA, Colonna M (2001) TREM-1 amplifies inflammation and is a crucial mediator of septic shock. Nature 410: 1103–7. 11323674 10.1038/35074114

[38] Millo JL, Schultz MJ, Williams C, Weverling GJ, Ringrose T, et al (2004) Compartmentalisation of cytokines and cytokine inhibitors in ventilator-associated pneumonia. Int Care Med 30: 68–74. 10.1007/s00134-003-2060-0 14634726

[39] Gomez-Pina V, Soares-Schanoski A, Rodriguez-Rojas A, Del Fresno C, Garcia F, et al (2007) Metalloproteinases shed TREM-1 ectodomain from lipopolysaccharide-stimulated human monocytes. J Immunol 179: 4065–73. 17785845 10.4049/jimmunol.179.6.4065

[40] Hartog CM, Wermelt JA, Sommerfeld CO, Eichler W, Dalhoff K, et al (2003) Pulmonary matrix metalloproteinase excess in hospital-acquired pneumonia. Am J Respir Crit Care Med 167: 593–8. 12588713 10.1164/rccm.200203-258OC

[41] Baselski V, Klutts JS (2013) Quantitative cultures of bronchoscopically obtained specimens should be performed for optimal management of Ventilator-Associated Pneumonia. J Clin Microbiol 51(3): 740–4. 23284021 10.1128/JCM.03383-12 PMC3592072

[42] Gibot S, Béné MC, Noel R, Massin F, Guy J, et al (2012) Combination biomarkers to diagnose sepsis in the critically ill patient. 186: 65–71. 10.1164/rccm.201201-0037OC 22538802

[43] Zethelius B, Berglund L, Sundstrom J, Ingelsson E, Basu S, et al (2008) Use of multiple biomarkers to improve the prediction of death from cardiovascular causes. N Engl J Med 358: 2107–2116. 18480203 10.1056/NEJMoa0707064

