# Supplementary material for: A Biomarker Panel (Bioscore) Incorporating Monocytic Surface and Soluble TREM-1 Has High Discriminative Value for Ventilator-Associated Pneumonia: A Prospective Observational Study
Source: PLoS One. 2014 Oct 7;9(10):e109686. doi: 10.1371/journal.pone.0109686 (PMC4188746; doi:10.1371/journal.pone.0109686)
Supplement: Table S1 — BALF bacterial growth in patient groups. The table shows the BALF microbiological results with patients of CPIS >6, CPIS <6 and CPIS = 6. CPIS = Clinical pulmonary infection score. VAP = Ventilator-associated pneumonia (DOC) [file pone.0109686.s001.doc]

**Table S1. BALF bacterial growth in patient groups**

| **Patient group** | **Number of patients** | **BALF bacterial growth** |
| --- | --- | --- |
| CPIS > 6 (VAP) | 20 | ALL mod to heavy |
| CPIS < 6 (non-VAP) | 33 | ALL none or light |
| CPIS 6 | 7 | ALL mod to heavy |
