# Supplementary material for: A Biomarker Panel (Bioscore) Incorporating Monocytic Surface and Soluble TREM-1 Has High Discriminative Value for Ventilator-Associated Pneumonia: A Prospective Observational Study
Source: PLoS One. 2014 Oct 7;9(10):e109686. doi: 10.1371/journal.pone.0109686 (PMC4188746; doi:10.1371/journal.pone.0109686)
Supplement: Materials and Methods S1 — Supporting information on study participants, biomarker measurement, biomarker panel construction and validation. (DOC) [file pone.0109686.s002.doc]

**On line data supplement**

**Materials and Methods**

**Study participants**

Exclusion criteria were patient refusal, HIV-1, Hepatitis B and C infection, tuberculosis, pregnancy, bleeding diathesis or pneumothorax. Informed, witnessed and written assent was obtained from a relative or designated carer for all ventilated patients. Written consent was obtained from all day case bronchoscopy patients.

VAP definition

VAP was defined as a CPIS>5 with positive semi-quantitative (SQ) microbiology (moderate or heavy growth >2+). Of the 27 patients with VAP, seven had a CPIS of 6. By the original CPIS definitions, they would have been classified as non-VAP. However, all of the seven patients had BALF SQ of moderate to heavy growth. Therefore, from a clinical perspective, they could and should be considered VAP, as we have done. Furthermore, all of the 33 cases classified as non-VAP (CPIS<6) had SQ of none or light growth (1+) (table S1).

We have also examined the cytokine data for the cases of CPIS >6, CPIS 6 and CPIS <6. The mean levels of all the seven biomarkers mTREM-1 (BALF/blood ratio), mCD11b (BALF/blood ratio), sTREM-1 (BALF), IL-1 beta (BALF), IL-8 (BALF), IL-6 (blood) and CRP (blood) in the cases with CPIS 6 were all in keeping with those cases of CPIS >6 as opposed to CPIS <6.

**Sampling, and processing of BAL fluid (BALF) and blood**

Paired venous blood and BALF samples were obtained from all study participants. In the Intensive Care Unit, bronchoscopy was performed by one of two experienced bronchoscopists, in sedated patients using an Olympus bronchoscope [BF-IT260] (Olympus, Tokyo, Japan). Patients were either off antibiotics, or were lavaged prior to a change of antibiotic. A bronchial segment corresponding to the new focal CXR shadowing was identified and lavaged, or the segment from where purulent secretions were most apparent. If there was no such indication of the site of new infection, or in the case of non-VAP patients, the right middle lobe was identified for lavage. In all ventilated patients, standard bronchoscopic techniques were used. One hundred and fifty ml of 0.9% saline was used for BAL and the initial 20ml of bronchial sample discarded. Half the sample was sent for microbiological analysis and the remainder filtered through sterile gauze to remove sputum, prior to processing in the immunology laboratory.

**Analysis of cellular inflammatory markers**

Monoclonal antibodies used in this study included fluorescein isothiocyanate (FITC)-conjugated anti-CD62L, FITC-conjugated anti-CD16, phycoerythrin (PE)-conjugated antiCD11b, phycoerythrin Texas red (ECD)-conjugated anti-CD45, phycoerythrin-cyanin red 5.1 (PC5)-conjugated anti-CD14, FITC and PE-conjugated IgG isotype controls (Beckman Coulter, High Wycombe, UK), PE-conjugated anti-TREM-1 (R&D Systems Minneapolis, USA), PE-conjugated IgG isotype control for TREM-1 (Pharmingen, Oxford, UK).

Cell acquisition was performed on a Cytomics FC500 flow cytometry (Beckman Coulter) and at least 5000 monocytes/neutrophils in blood and 2000 monocyte/neutrophil cells were analysed. ‘Flowcheck’ beads (Beckman Coulter) were used to calibrate flow cytometer instrumentation settings and to control for spectral overlap. FlowJo software (BD BioSciences, Ontario Canada) was used for data analysis.

**Quantification of cytokines and inflammatory mediators**

Concentrations of sTREM-1, IL-1β, IL-6 and IL-8 were measured in duplicate by ELISA (R&D Systems Minneapolis, USA) in plasma and BALF samples according to manufacturer’s instructions. Procalcitonin was measured quantitatively using a Brahms PCT mini-VIDAS machine (Biomerieux, Hampshire, UK) in plasma and BALF samples. White cell counts and CRP were measured by the National Health Service (NHS) diagnostic laboratories as part of patients’ routine clinical care.

A database was constructed with each case and the following biomarkers:

**Blood**

(i) CRP

(ii) WCC

(iii) sTREM-1

(iv) IL-6

(v) PCT

(vi) mTREM-1

(vii) nTREM-1

(viii) mCD11b

(ix) nCD11b

(x) mCD62L (L-selectin)

(xi) nCD62L (L-selectin)

**BALF**

(xii) sTREM-1

(xiii) IL-1β

(xiv) IL-6

(xv) IL-8

(xvi) PCT

(xvii) mTREM-1

(xviii) nTREM-1

(xix) mCD11b

(xx) nCD11b

(xxi) mCD62L (L-selectin)

(xxii) nCD62L (L-selectin)

**BALF/blood ratio**

(xxiii) sTREM-1

(xxiv) IL-6

(xxv) PCT

(xxvi) mTREM-1

(xxvii) nTREM-1

(xxviii) mCD11b

(xxix) nCD11b

(xxx) mCD62L (L-selectin)

(xxxi) nCD62L (L-selectin)

For the biomarkers IL-1β and IL-8, blood levels were below detectable limits. Therefore neither the blood nor the BALF/blood ratios were reported. Similarly, WCC and CRP were measured in the blood only and therefore BALF and BALF/blood ratios were not reported.

**Group classification**

In the study, we recruited patients with VAP, ventilated without the presence of infection and patients ventilated with non-pulmonary infection (e.g. confirmed central line or abdominal sepsis). In the total of 33 patients ventilated without VAP, 18 had no infection present and 15 had non-pulmonary sepsis. The bacteria identified were in the patients with non-pulmonary sepsis. The BALF and blood biomarker data was analysed for each group separately. There was a significant difference between the VAP group and the other two non-ventilated groups. However, there were no numerical nor statistical differences between the BALF biomarker data (or BALF SQ microbiological data – all no or light growth) in the ventilated non-infected group and the ventilated non-pulmonary sepsis group. Therefore it was felt justified to combine these 2 groups into a single ventilated non-VAP group (comprising patients without pulmonary infection or non-pulmonary infection).

**Construction of the biomarker panel**

Fisher discriminant analysis (FDA) was used to construct a panel of putative biomarkers. This statistical technique constructs a linear set of functions derived from the markers that accurately classifies patients into a categorical label, in this case a patient group (i.e VAP, non-VAP, NVC). This differs from regression analysis where the classification dependent variable is numerical. FDA assumes a normal population distribution. We felt that the use of a pool of greater than 20 biomarkers would allow this condition to be fulfilled. FDA maximizes the discrimination between the groups, whilst minimizing differences in variance. When samples are described by two variables on x and y axes, FDA creates a vector that the variables are then mapped to. This vector increases the scatter between the groups (class separation), but minimize scatter within each group (variance). This analysis is then repeated for multiple variables to determine the optimal combination of ‘vectors’ between them.

Further analysis using Cox regression (which does not mandate a parametric distribution), was conducted by Professor Athol Wells. The results were the same as those obtained from FDA. Finally, stepwise forward linear regression analysis was conducted by Dr. Panagiotis Pantelidis. In this method, a panel is constructed by determining an optimal first marker, then sequentially adding further markers until there is no further improvement in diagnostic accuracy. These results also agreed with those derived from FDA.

**Biomarker panel validation**

Following construction of a biomarker panel, it can be tested with data acquired from new patients to determine its efficacy (validation). However, given finite time and resources, additional recruitment of new patients was not possible.

Therefore, we conducted two further internal validity analyses. The first analysis comprised the ‘leave one out’ classification (cross-validation). In this, outlier effects are determined and excluded. Each of the 91 patients was serially excluded from the patient pool in turn and the Fisher analysis re-conducted. With strong outlier effects, high variability in the panel would be seen. This was not the case.

The second analysis involved cross-validation by splitting the data into a training (60%) and validation cohort (40%). This technique has previously been used by our research group to successfully validate a biomarker panel [21]. With this method, 60% of patients are randomly chosen to construct the panel using FDA. In effect, this creates new classification function coefficients for each analyte in the biomarker panel [22-24]. The panel functions are then applied to the remaining 40% of patients as a test cohort. This simulates the effect of recruiting new patients not used in the original panel generation. Repetition of this analysis ten times allows a summary panel utility to be determined. This validation technique advantageously tests the panel on ‘new data’ but at the expense of constructing the initial panel with a smaller (60%) subset of patients and the attendant risk of greater result variability.
